# Parathyroid hormone may be an early predictor of low serum hemoglobin concentration in patients with not advanced stages of chronic kidney disease

**DOI:** 10.1007/s40620-014-0129-1

**Published:** 2014-08-12

**Authors:** Domenico Russo, Luigi Morrone, Biagio Di Iorio, Michele Andreucci, Maria Grazia De Gregorio, Carmela Errichiello, Luigi Russo, Francesco Locatelli

**Affiliations:** 1 Department of Nephrology, University of Naples “FEDERICO II”, Via S. Pansini, 5, 80131 Naples, Italy; 2 Department of Nephrology, “G. RUMMO” Hospital, Benevento, Italy; 3 Department of Nephrology, “A. LANDOLFI” Hospital, Solofra, Avellino Italy; 4 Department of Health Sciences, University of Catanzaro, Catanzaro, Italy; 5 Department of Nephrology, Alessandro Manzoni Hospital, Lecco, Italy

**Keywords:** Parathyroid hormone, Hemoglobin, Anemia, Chronic kidney disease

## Abstract

**Background:**

Parathyroid hormone (PTH) has been associated with anemia only in dialysis patients with severe hyperparathyroidism. Whether an association between PTH and hemoglobin also exists in patients with chronic kidney disease not on dialysis (CKD-patients) is still unclear. In this study we evaluated the association between PTH and hemoglobin in CKD-patients without severe secondary hyperparathyroidism.

**Methods:**

Hospitalized patients and outpatients (N = 979) were retrospectively evaluated and categorized according to PTH quartile and serum hemoglobin (<12.0, <11.0, <10.0 g/dl). Gender, diabetes, glomerular filtration rate (GFR), hemoglobin, PTH, markers of mineral metabolism, inflammation, iron status and nutrition were variables of adjustment in univariate and multivariate analysis.

**Results:**

An inverse association (p = 0.001) was observed between PTH and hemoglobin in patients as a whole, in diabetics, and in patients with GFR ≤60 ml/min. PTH was the single predictor of low hemoglobin in patients as a whole (unstandardized beta −2.12; p = 0.005), in diabetics (unstandardized beta −8.86; p = 0.007) and in patients with GFR ≤60 ml/min (unstandardized beta −2.52; p = 0.006). For each increase of quartile of PTH the risk of having hemoglobin level <10.0 mg/dl was more than doubled [hazard ratio (HR) 2.79, 95 % confidence interval (CI) 2.00–3.88; p = 0.001]. The receiver operating characteristic curve showed that PTH ≥122 pg/ml had 67 % sensitivity and 75 % specificity in predicting hemoglobin level <10.0 g/dl with area under the curve of 0.758 (95 % CI 0.73–0.78).

**Conclusions:**

This study shows a significant inverse association between PTH and hemoglobin levels across the whole spectrum of non-dialysis CKD and a doubled risk of having serum hemoglobin <10.0 mg/dl in the absence of severely deranged PTH concentration. These findings may have clinical relevance in ascertaining the cause of unexplained low hemoglobin levels in CKD-patients.

## Introduction

In past experimental studies it was observed that synthesis of endogenous erythropoietin, formation of erythroid progenitors, and survival of red cells were reduced by a high PTH [1–3]. Similarly, negative effects of very high serum levels of PTH on serum hemoglobin were observed in patients on dialysis. In fact, improved serum concentration of hemoglobin, need for lower doses of erythropoietin stimulating agents and reduced fibrosis of bone marrow were attained after either better control of secondary hyperparathyroidism or parathyroidectomy [4–12].

Whether the association between PTH and hemoglobin exists in patients with chronic kidney disease not on dialysis (CKD-patients) has been little evaluated. The aim of the present study was to assess whether there is an association between PTH and hemoglobin in CKD-patients without severely deranged serum PTH concentrations and who did not undergo previous therapy with erythropoietin stimulating agents and/or vitamin D. Confirmation of this association may have clinical relevance in assessing the cause of unexplained low hemoglobin level in CKD-patients.

## Subjects and methods

This is a retrospective observational cohort study performed in hospitalized patients and outpatients who had been referred to a single Nephrology Unit from year 2002 to year 2009. The study was approved by the local Institutional Review Board.

Criteria for evaluation were age >18 years, at least 6 months of follow-up prior to the collection of pertinent data, and two assays of PTH and hemoglobin performed by the same laboratory. Exclusion criteria were: stage 5 chronic kidney disease requiring dialysis treatment, rapidly progressive glomerulonephritis, polycystic kidney disease, past or recent therapy with iron, erythropoietin stimulating agents, or vitamin D sterols, presence of bleeding and/or need for blood transfusion in the previous medical history, and current treatment with warfarin that may cause unapparent blood loss.

Patients underwent full clinical examination, medical history and routine blood chemistry. Diabetic patients were defined as those on regular use of insulin or oral hypoglycemic drugs. Patient characteristics and routine chemistry were stratified according to quartiles of serum PTH concentration. Presence of anemia was established on the basis of three cut-off levels of serum hemoglobin; the first cut-off was serum hemoglobin <12.0 g/dl, that has been indicated as the lower normal level of hemoglobin for the diagnosis of anemia in adult females with CKD [13]; we arbitrarily extended this level to the male population. The second cut-off was serum hemoglobin <11.0 g/dl, that is regarded as the threshold for starting erythropoietin stimulating agents in CKD-patients [14–16]. The third cut-off was serum hemoglobin <10.0 g/dl, since hemoglobin concentration of 10.0 g/dl has recently been suggested as the lowest adequate level of hemoglobin in European CKD-patients [17].

Analyses were performed on patients as a whole, and on two subgroups: those with diabetics, and those with glomerular filtration rate (GFR) ≤60 ml/min. The subgroup analysis was done since previous studies which have evaluated the epidemiology of anemia associated with chronic renal insufficiency and in diabetics [18–20] did not take into account levels of PTH as a potential factor responsible for reduced hemoglobin levels.

Blood samples were collected in the morning in fasting state. Variables assessed were: glucose, urea, hemoglobin, iron, ferritin, transferrin saturation (TSAT %), calcium, phosphorus, intact parathyroid hormone (i-PTH), 24/h urinary protein excretion, alkaline phosphatase, uric acid, homocysteine, C-reactive protein (hs-CRP), triglycerides, total cholesterol, high-density lipoprotein cholesterol (HDL-cholesterol) and low-density lipoprotein cholesterol (LDL-cholesterol).

Body mass index (BMI) was computed as weight (in kilograms) divided by the square of the height (in meters). Kidney function was assessed by 24/h-measured creatinine clearance and reported as GFR. i-PTH was assayed by the chemiluminescent immunometric method. Hs-CRP was assayed by the immunoturbidimetric method. LDL-cholesterol was calculated using the Friedewald equation. TSAT was calculated dividing serum iron and total iron-binding capacity, multiplied by 100.

### Statistics

Continuous data are reported as median and interquartile range. Frequency data are given as numbers and percentages. Normal distribution of data was tested using the Shapiro–Wilk test. Skewed variables were log-transformed to improve normality in regression models. Univariate correlations between continuous variables were analyzed by Pearson’s test. Variables significantly associated with hemoglobin at univariate analysis were used for multivariate linear regression. Hemoglobin concentration was used as the dependent variable in multivariate linear regression models to identify factor(s) independently associated with hemoglobin in patients as a whole, in those with diabetes, and in those with GFR ≤60 ml/min. Presence of multicollinearity was checked and collinear variables were excluded from multivariate analysis. Logistic regression was used to predict to what extent incremental quartiles of PTH increased the risk of having serum hemoglobin levels <10.0 mg/dl. A receiver operating characteristic (ROC) curve was generated to assess sensitivity and specificity of PTH in predicting serum hemoglobin levels <10 mg/dl.

Values of p < 0.05 were considered as statistically significant.

Data were analyzed with the Statistical Package for the Social Sciences (SPSS) version 19 (Chicago, IL, USA).

## Results

The initial number of screened patients was 1,093, of which 114 were non eligible. Causes for ineligibility were: chronic lung disease (n = 35), inability to give reliable personal medical history (n = 30), severe liver disease (n = 25), chronic therapy with nonsteroidal anti-inflammatory drugs (NSADs) (n = 15), and hemorrhoids (n = 9). The final analysis was performed on data of 979 patients.

The primary renal diagnoses were: hypertensive nephropathy (26 %), glomerulonephritis (16 %), diabetes (11 %), ischemic nephropathy (6 %), minor urinary abnormalities (4 %), interstitial nephritis (4 %), and CKD of unknown cause (34 %). The majority of the study population did not have severe derangement of PTH levels (upper normal limit for our laboratory 75 pg/ml); in detail, PTH >150 pg/ml was found in 207 (21 %) patients while PTH >1,000 pg/ml was found in 3 (0.3 %) patients.

Clinical characteristics and biochemistry of patients were evaluated in aggregate and according to quartiles of PTH; the results are shown in Table 1. Patients in the highest quartile of PTH were more likely to have reduced GFR, serum albumin concentration, total cholesterol and LDL-cholesterol; in addition, they were more likely to have increased serum concentration of fibrinogen and homocysteine and 24/h urinary protein excretion.

**Table 1:** Clinical characteristics and biochemistry of patients as a whole and of patients subdivided according to quartiles of parathyroid hormone

	All patients	1st quartile PTH (≤50 pg/ml)	2nd quartile PTH (51–69 pg/ml)	3rd quartile PTH (70–132 pg/ml)	4th quartile PTH (≥133 pg/ml)	P across quartiles
No. patients	979	245	245	244	245	
Age (years)	56.8 (48.0–66.0)	54.0 (44.0–64.0)	53.0 (48.0–60.0)	62.0 (50.0–70.0)	59.0 (50.0–73.0)	0.000
Male (%)	658 (67)	175 (71.8)	153 (62.9)	165 (66.8)	165 (67.3)	0.166
Diabetes (%)	243 (24.8)	51 (21)	59 (24)	71 (29)	61 (25)	0.330
Body mass index (kg/m^2^)	26.7 (24.1–30.0)	26.7 (24.0–30.0)	26.9 (24.2–32.0)	26.1 (23.8–29.9)	25.9 (24.0–29.2)	0.547
Glomerular filtration rate (ml/min)	36.0 (28.0–67.0)	74.0 (49.5–110.0)	30.5 (28.4–60.9)	33.8 (28.4–49.0)	29.0 (20.0–36.0)	0.000
Hemoglobin (g/dl)	12.5 (11.0–14.0)	14.0 (12.8–15.3)	12.5 (11.0–13.7)	12.5 (11.0–13.6)	11.3 (10.5–12.2)	0.000
Iron (µg/dl)	76.0 (57.0–97.0)	82.0 (61.0–103.0)	82.5 (64.0–103.5)	66.0 (54.0–92.0)	68.0 (46.5–92.5)	0.000
Ferritin (ng/ml)	105.0 (54.0–187.0)	107.0 (50.5–200.5)	104.5 (49.3–175.3)	100.0 (52.0–169.0)	100.0 (60.0–168.0)	0.198
Transferrin (mg/dl)	302 (266–343)	312 (272–356)	298 (266–334)	300 (264–340)	295 (253–331)	0.045
Transferrin saturation (%)	18 (14–23)	19 (14–23)	21 (16–25)	17 (13–20)	19 (13–24)	0.019
Calcium (mg/dl)	9.4 (9.0–9.8)	9.6 (9.2–9.8)	9.6 (9.3–10.0)	9.4 (9.0–9.9)	9.0 (8.5–9.4)	0.000
Phosphorus (mg/dl)	4.0 (3.3–4.8)	3.6 (3.1–4.1)	4.6 (3.5–5.0)	3.8 (3.2–4.7)	4.4 (3.8–5.3)	0.000
Alkaline phosphatase (IU/l)	77 (60–98)	70 (57–91)	73 (59–90)	84 (66–106)	93 (69–122)	0.000
Proteinuria (mg/24 h)	621 (210–1550)	525 (205–1,330)	330 (174–1,440)	675 (217–1,380)	1152 (357–2,407)	0.000
C-reactive protein (mg/dl)	6.0 (2.0–9.0)	1.1 (1.1–5.7)	8.0 (5.0–10.0)	7.0 (2.0–10.0)	4.5 (2.0–7.0)	0.007
Uric acid (mg/dl)	6.1 (5.1–7.2)	5.7 (4.8–6.8)	5.8 (4.7–6.7)	6.6 (5.6–7.5)	6.8 (5.8–7.9)	0.000
Fibrinogen (mg/dl)	364.0 (300.0–455.0)	330.0 (276.0–431.0)	353.0 (293.0–426.0)	372.0 (308.0–450.0)	399.0 (343.0–498.0)	0.000
Serum albumin (mg/dl)	4.2 (3.8–4.6)	4.4 (4.2–4.7)	4.4 (4.1–4.6)	4.2 (3.8–4.6)	3.9 (3.5–4.3)	0.000
Homocysteine (µg/l)	18.3 (14.0–23.8)	16.0 (12.0–21.3)	17.2 (14.3–21.1)	21.5 (16.5–27.0)	26.5 (20.0–35.2)	0.000
Total cholesterol (mg/dl)	179 (151–210)	190 (162–226)	187 (158–212)	175 (147–200)	166 (139–191)	0.000
Triglycerides (mg/dl)	122 (93–176)	122 (92–172)	109 (81–146)	125 (96–174)	128 (99–190)	0.048
HDL-cholesterol (mg/dl)	48 (38–58)	48 (38–59)	50 (39–63)	48 (40–56)	47 (35–56)	0.225
LDL-cholesterol (mg/dl)	103 (82–129)	113 (91–142)	107 (84.8–131)	100 (77–118)	98 (82–115)	0.000
Hemoglobin <12.0 mg/dl (%)	399 (40.8)	33 (13.5)	105 (42.8)	99 (40.5)	161 (65.5)	0.000
Hemoglobin <11.0 mg/dl (%)	225 (23)	13 (5.3)	62 (25.4)	55 (22.5)	91 (37.1)	0.000
Hemoglobin <10.0 mg/dl (%)	103 (10.5)	8 (3.5)	3 (1.5)	31 (12.5)	60 (24.5)	0.000

At univariate analysis serum hemoglobin was significantly (p = 0.001) associated with: age (r = −0.21), gender (r = −0.20), diabetes (r = −0.27), PTH (r = −0.42), serum calcium (r = −0.47), serum phosphorus (r = 0.60), GFR (r = 0.32), transferrin saturation (r = 0.19), CRP (r = −0.34), homocysteine (r = −0.26), serum albumin (r = 0.49), and serum cholesterol (r = 0.24); serum hemoglobin was not associated with ferritin (r = 0.10; p = 0.08) or BMI (r = 0.04; p = 0.30).

**Fig. 1:**
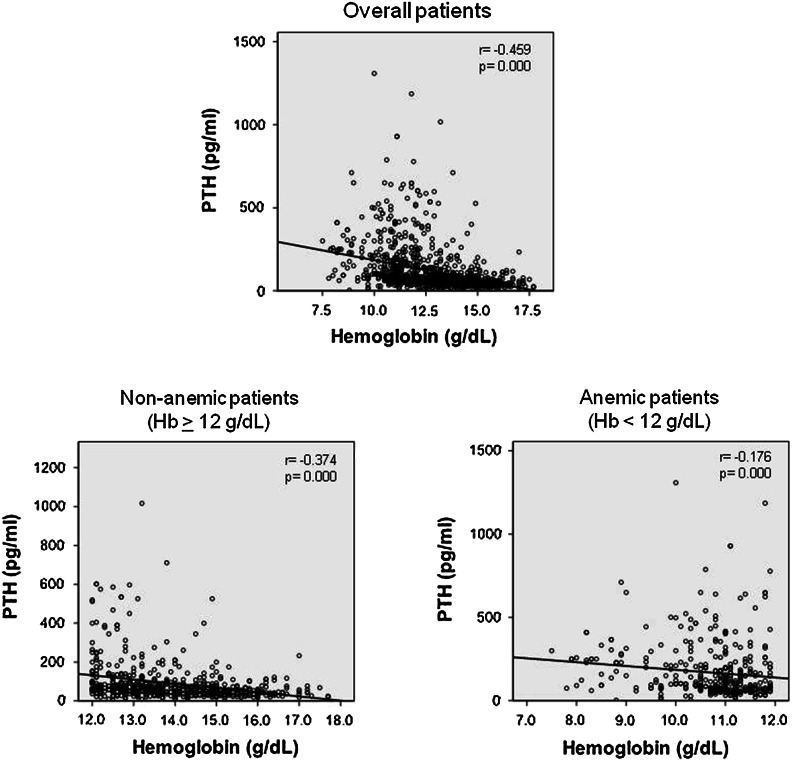
Association between PTH and hemoglobin serum concentration in patients as a whole, and in anemic (serum hemoglobin <12.0 g/dl) and non-anemic patients (serum hemoglobin ≥12.0 g/dl)

The inverse association between PTH and hemoglobin is shown in Fig. 1. A significant association was observed in patients as a whole (r = −0.459; p = 0.000), in patients with serum hemoglobin <12.0 g/dl (r = −0.176; p = 0.000), and in patients with serum hemoglobin >12.0 g/dl (r = −374; p = 0.01).

The results of multivariate linear regression analysis in patients as a whole are reported in Table 2. There was collinearity of fibrinogen and homocysteine with CRP; thus fibrinogen and homocysteine were excluded from the final model. In the final model, high PTH was the only significant predictor of low hemoglobin (unstandardized coefficients beta −2.12; p = 0.005). Of interest, we found a significant interaction between log-PTH and GFR (p = 0.006); despite this interaction, PTH remained the single significant (p = 0.000) predictor of low hemoglobin.

**Table 2:** Multiple regression analysis with serum hemoglobin concentration as the dependent variable in patients as a whole

	Unstandardized coefficients B	Std. error	t	p	95.0 % confidence interval for B	
					Lower	Upper
Age	−0.01	0.02	−0.79	0.433	−0.05	0.02
Gender	−0.77	0.48	−1.60	0.116	−1.74	0.20
Diabetes	−0.11	0.48	−0.22	0.824	−1.08	0.87
GFR	0.00	0.01	0.07	0.947	−0.02	0.02
PTH^a^	−2.12	0.73	−2.91	0.005	−3.59	−0.66
Serum calcium	−0.02	0.41	−0.05	0.957	−0.84	0.79
Serum phosphorus	−0.50	0.33	−1.54	0.131	−1.16	0.16
TSAT	0.03	0.03	0.98	0.332	−0.03	0.08
Albumin	0.17	0.50	0.35	0.731	−0.84	1.18
Cholesterol	0.01	0.01	2.00	0.051	0.00	0.02
PCR^a^	−0.57	0.66	−0.86	0.395	−1.89	0.76

Due to presence of collinearity log-ferritin, fibrinogen, homocysteine, BMI were excluded from the final model

*GFR* glomerular filtration rate (as 24/h measured creatinine clearance), *PTH* parathyroid hormone, *PCR* C-reactive protein, *TSAT* transferrin saturation

^a^Log transformed variable

**Fig. 2:**
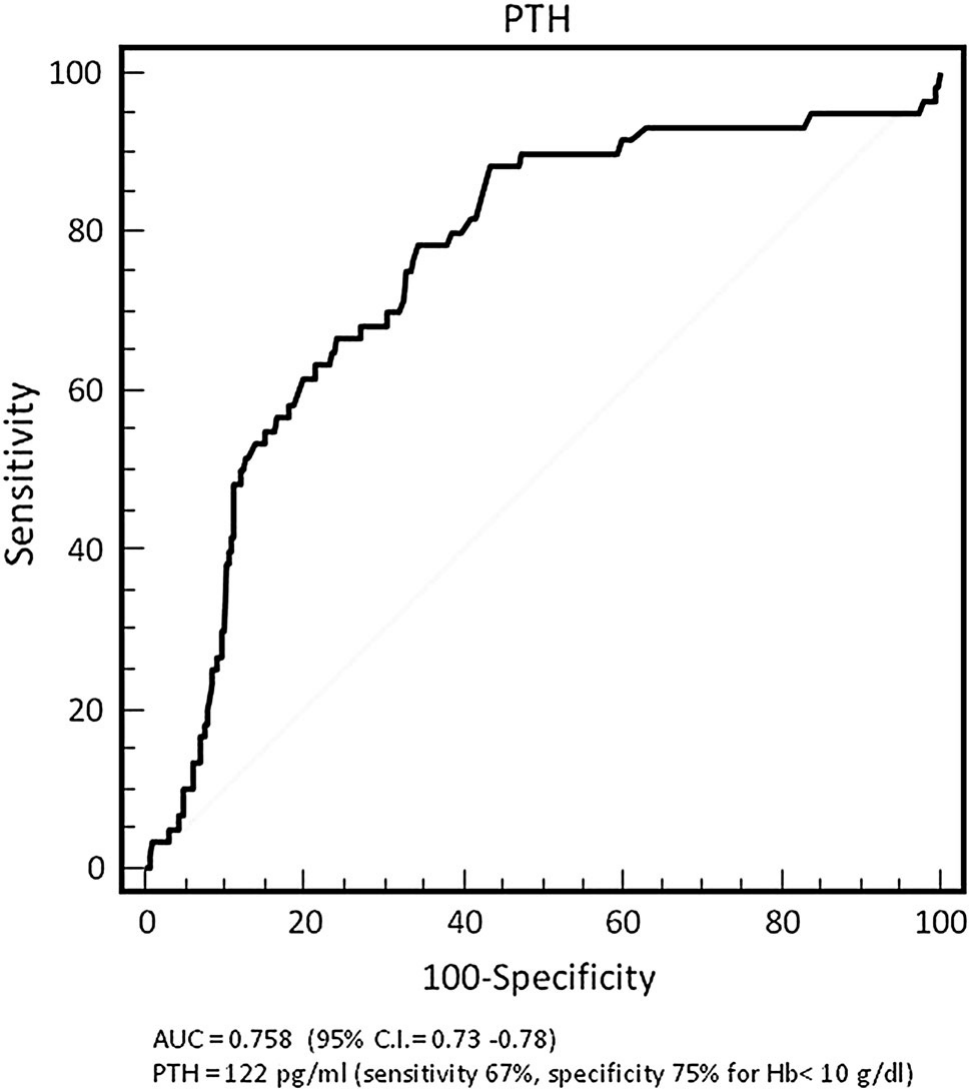
Receiver operating characteristic curve for sensitivity and specificity of PTH in predicting serum hemoglobin concentration <10.0 mg/dl in patients as a whole

The association between PTH and hemoglobin levels in patients as a whole is reported in Table 1. There was an inverse association between PTH and hemoglobin levels (p = 0.001). In logistic regression, for each increase of quartile of PTH the risk of having hemoglobin levels <10.0 mg/dl was more than doubled [hazards ratio (HR) 2.79, 95 % confidence interval (CI) 2.00–3.88, p = 0.001]. The ROC curve is shown in Fig. 2. PTH ≥122 pg/ml had a sensitivity of 67 % and a specificity of 75 % in predicting hemoglobin level <10.0 g/dl with area under the curve of 0.758 (95 % CI 0.73–0.78).

Results of multivariate linear regression analysis in diabetics and in patients with GFR ≤60 ml/min are reported in Tables 3 and 4, respectively. The collinearities found in patients as a whole were found also in diabetic patients and in those with GFR ≤60 ml/min; for these subgroups, therefore, the final multivariate linear regression model had similar variables as for the final model of patients as a whole. High PTH was confirmed as the single predictor of low serum hemoglobin concentration in diabetic patients (unstandardized coefficients beta −8.86, p = 0.007) and in patients with GFR ≤60 ml/min (unstandardized coefficients beta −2.52, p = 0.006).

**Table 3:** Multiple regression analysis with serum hemoglobin concentration as the dependent variable in diabetics (N = 165)

	Unstandardized coefficients B	Std. error	t	p	95.0 % confidence interval for B	
					Lower	Upper
Age	−0.10	0.08	−1.32	0.244	−0.31	0.10
Gender	−0.65	1.09	−0.60	0.578	−3.45	2.15
GFR	−0.01	0.02	−0.66	0.536	−0.06	0.04
PTH^a^	−8.86	2.00	−4.43	0.007	−14.00	−3.72
Serum calcium	2.58	0.95	2.71	0.042	0.13	5.02
Serum Phosphorus	−0.43	0.52	−0.83	0.445	−1.77	0.91
TSAT	−0.01	0.03	−0.26	0.809	−0.08	0.07
Albumin	−3.49	1.20	−2.91	0.033	−6.58	−0.41
Cholesterol	0.03	0.01	2.68	0.044	0.00	0.05
PCR^a^	2.26	1.28	1.77	0.138	−1.03	5.55

Due to presence of collinearity log-ferritin, fibrinogen, homocysteine, BMI were excluded from the final model

*GFR* glomerular filtration rate (as 24/h measured creatinine clearance), *PTH* parathyroid hormone, *PCR* C-reactive protein, *TSAT* transferrin saturation

^a^Log transformed variable

**Table 4:** Multiple regression analysis with serum hemoglobin concentration as dependent variable in patients with GFR <60 ml/min (N = 790)

	Unstandardized coefficients B	Std. error	t	p	95.0 % confidence interval for B	
					Lower	Upper
Age	−0.02	0.02	−1.15	0.258	−0.07	0.02
Gender	−0.50	0.76	−0.66	0.515	−2.06	1.05
Diabetes	−0.39	0.64	−0.61	0.546	−1.70	0.92
GFR	0.01	0.02	0.58	0.565	−0.04	0.06
PTH^a^	−2.52	0.86	−2.94	0.006	−4.27	−0.77
Serum calcium	0.22	0.47	0.46	0.648	−0.74	1.17
Serum phosphorus	−0.60	0.44	−1.37	0.181	−1.50	0.30
TSAT	−0.42	0.87	−0.48	0.633	−2.21	1.37
Albumin	0.41	0.78	0.52	0.607	−1.19	2.01
Cholesterol	0.01	0.01	1.08	0.288	−0.01	0.03
PCR^a^	0.05	0.05	1.02	0.317	−0.05	0.15

*GFR* glomerular filtration rate (as 24/h measured creatinine clearance), *PTH* parathyroid hormone, *PCR* C-reactive protein, *TSAT* transferrin saturation

^a^Log transformed variable

The majority of anemic patients were found in the highest quartile of PTH whatever the level of hemoglobin used for the definition of anemia (Table 1). The percentage of anemic patients was even higher in diabetics and in patients with GFR ≤60 ml/min. Indeed, percentage of anemic diabetic patients was 62, 37, and 21 % at levels of hemoglobin <12.0, <11.0, and <10.0 g/dl. The percentage of anemic patients with GFR ≤60 ml/min was 84, 43, and 31 % at levels of hemoglobin <12.0, <11.0, and <10.0 g/dl.

## Discussion

Parathyroid hormone has been associated to anemia in the past. However, this association was ascertained in patients on chronic dialysis treatment with long-lasting secondary hyperparathyroidism and very high serum concentration of PTH [4–12]. In the present study, the inverse association between PTH and serum hemoglobin concentration was observed across the whole spectrum of non-dialysis CKD without severe secondary hyperparathyroidism. This finding may be relevant in clinical practice.

Serum concentrations of PTH that may be regarded as not relevant in everyday care of CKD-patients were a predictor of low hemoglobin concentration. The progression of PTH from the 1st to the 4th quartile was associated to a progressive significant decrease of hemoglobin concentration. The increase of each PTH quartile was associated to more than a doubled risk of having hemoglobin levels <10.0 mg/dl.

The association between PTH and hemoglobin was independent of factors that are commonly related to anemia in dialysis patients; age, gender, iron status, biomarkers of inflammation and nutrition were, in fact, not predictors of hemoglobin concentration. Interestingly, the negative association of PTH with hemoglobin was independent of reduced renal function, indicating that PTH is a marker of low hemoglobin independently of level of GFR.

The data observed in the present study are different from those of previous published reports. In CKD-patients who had been evaluated for a community-based screening program a significant positive association between PTH and hemoglobin was observed only in diabetics [21]. In the present study, we observed a negative association between PTH and hemoglobin levels both in diabetics and in patients with GFR ≤60 ml/min. These conflicting data may be possibly explained by the following factors. In the community screening study, diabetics represented half of study population; the majority of the participants were female (69.2 %) and were 70 years old; all participants had GFR <60 ml/min; cancer was present in 20 % of the population; iron parameters were not assessed; biochemical parameters inclusive of PTH and hemoglobin were assayed in several laboratories and not in standardized conditions of blood sampling and fasting state.

The percentage of anemic patients recorded in the present study should be highlighted. Indeed, categorizing as anemic our patients on the basis of three cut-off levels of hemoglobin, anemia was relevant whatever hemoglobin level was taken into the account. Indeed, in the whole cohort, the percentage of anemic patients ranged from 41 to 11 % considering as cut-off the levels of hemoglobin lower than 12.0, 11.0 and 10.0 g/dl. In addition, the percentage of patients with anemia was always higher in patients in the highest quartile of PTH whatever the level of serum hemoglobin concentration used for the definition of anemia.

In our opinion, the data of the present study may be clinically relevant in ascertaining the cause of unexplained low hemoglobin level in CKD-patients without severe secondary hyperparathyroidism. In the presence of reduced serum hemoglobin concentration in patients with biomarkers of inflammation and nutrition, the rational therapeutic option is to initiate therapy with erythropoietin stimulating agents. On the basis of data observed in the present study, therapy with erythropoietin stimulating agents would have been started in 20 % of patients even using the most restrictive level of serum hemoglobin (e.g. hemoglobin <10.0 g/dl). More likely it should be advisable to take into the account the serum PTH even when its concentration seems not severely deranged. In conjunction to PTH assay the serum vitamin D assay should also be taken into account, that has been found to be associated to a decrease of hemoglobin and presence of anemia in CKD-patients [22].

The present study has several limitations. The design does not allow to establish causality but only an association between PTH and hemoglobin levels. We were also unable to determine the exact mechanisms explaining the association between PTH and serum hemoglobin levels. We cannot completely rule out residual confounding, although we adjusted for the common potential explanatory factors. Vitamin D levels were not assessed. Despite these limitations, our study has the strength of having examined a large population of adults with different degrees of CKD in a real-world clinical practice setting. Importantly, the patients did not receive previous erythropoietin stimulating agents or vitamins D; therefore the association between PTH and hemoglobin was evaluated without the confounding effects of these therapies.

Additional interventional studies are mandatory both to confirm the association between PTH and hemoglobin, and to assess whether better control of serum PTH may improve serum level of hemoglobin in CKD-patients.
